# Dietary sugar and Barrett’s esophagus

**DOI:** 10.1007/s10353-017-0494-9

**Published:** 2017-10-24

**Authors:** M. Riegler, I. Kristo, R. Asari, E. Rieder, S. F. Schoppmann

**Affiliations:** 1 Reflux Medical Vienna, Vienna, Austria; 2 Upper-GI Service, CCC-GET, University Clinic of Surgery, Vienna General Hospital — Medical University Vienna, Vienna, Austria

**Keywords:** Gastroesophageal reflux disease, Sweetened desserts/beverages, Anti-reflux surgery, Radiofrequency ablation

## Abstract

**Introduction:**

Barrett’s esophagus (BE) represents the premalignant morphology of gastroesophageal reflux disease (GERD). Evidence indicates a positive correlation between GERD vs. obesity and increased sugar consumption.

**Methods:**

Here we analyzed recently published data (2006–2017) on the role of dietary sugar intake for BE development (main focus year 2017).

**Results:**

Recent investigations found a positive association between obesity, hip waist ratio and dietary sugar intake and Barrett’s esophagus.

**Conclusion:**

Sugar intake positively associates with BE. A low carbohydrate diet should be recommended for persons with BE and GERD.

## Introduction

**Fig. 1:**
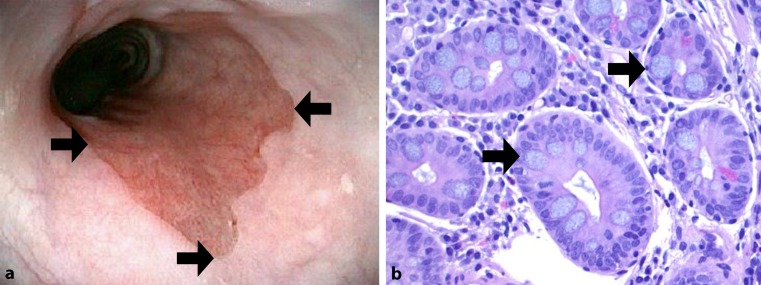
Antegrade endoscopic image of the esophagogastric junction (**a**). Note the presence of endoscopically visible columnar lined esophagus (*arrows*). Biopsies obtained from the junction contained columnar lined esophagus with goblet cells (*arrow*), the hallmark for Barrett’s esophagus (**b**). **a** Storz endoscopy technology; **b** H&E stain; courtesy of Prof Dr. Fritz Wrba, Vienna

Gastroesophageal reflux disease (GERD) affects 20–30% of the population [1–3]. In addition to the symptoms (heartburn, regurgitation, wheezing, asthma, etc.) [1–3], GERD may be complicated by Barrett’s esophagus (BE) [5–7]. The presences of biopsy-proven goblet cell containing intestinal metaplasia (IM) within columnar lined esophagus (CLE) defines BE ([1, 4, 5]; Fig. 1). As a reflux-induced tissue response, BE harbors an increased risk for the development of esophageal adenocarcinoma (annual risk ranges from 0.12–0.7%, mean 0.5%) [7]. Novel treatment options contribute to design effective cancer prevention strategies including radiofrequency ablation of BE and anti-reflux surgery [4–6].

Recent evidence highlights the association of GERD and BE with obesity and diet, i. e., sugar consumption [8–12]. Therefore, this paper aims to analyze recent data on the role of sugar intake for BE development.

## Methods

We analyzed recently published data (2006–2017) on the association between Barrett’s esophagus (BE) and dietary sugar intake, using PubMed (main focus of the analyses: year 2017). Statistics were not applied.

## Results

Our search assessed an analysis testing the correlation between lifestyle (nutrition) and Barrett’s esophagus (BE). The study by Li et al. [13] included two US community-based case–control studies: the Washington-based study of reflux disease (1997–2000) [11] and the Northern California-based epidemiology and incidence of BE study (2000–2005) [10]. Li et al. [13] pooled the data of the two studies and tested the effect of sugar consumption on the risk for BE development.

The pooled analysis compared the consumption of carbohydrate-containing food and beverages consumption of 472 BE-positive vs. 492 BE-negative controls (randomly matched BE-negative residents within the database) [13]. BE diagnosis required the histopathology of esophageal biopsies containing intestinal metaplasia (IM)-positive columnar lined esophagus (CLE). Dietary habits and carbohydrate consumption were assessed using a validated food frequency questionnaire and a detailed full spectrum catalog of twelve measures for sugar/starches intake including sugar components (free glucose, fructose, sucrose), added sugar, total sugar, starch, sweeteners, artificial sugars, glycemic load, nutrition with sweetened foods, and beverages. Sugar intake has been thus calculated and given in g/day for the year prior to BE diagnosis (cases) and interview (controls). Finally, data were compared vs. other factors including sex, race, body mass index (BMI), frequency of GERD symptoms, and total energy intake (kcal/day). Statistics applied odds ratios for logic regression for the assessment of risk associations [13].

The major finding of the study was that intake of sucrose, added sugar, and sweetened desserts/beverages was higher in BE-positive cases, vs. BE-negative controls (Table 1; [13]). Furthermore, following risk adjustment, risk for BE was increased 79%, and 71% among those in the highest vs. lowest quartiles of sucrose and added sugar intake, respectively [13]. Consumption of sweetened desserts and beverages associated with 71% increase in BE. In those with lower waist circumference, the association for BE risk was increased for sweetened desserts and beverages. Risk of short segment BE (≤3.0 cm) associated with increased intake of sucrose, total sugar, starch, total carbohydrate, glycemic load, sweetened desserts, and beverages. Such associations were not found for long segment BE (greater 3.0 cm). None of the other correlations and associations were statistically significant. Taken together, the study by Li et al. suggests a positive correlation between BE and sugar consumption [13].

**Table 1:** Sugar intake (g/day) in Barrett’s esophagus-negative and -positive individuals [13]

Sugar	Study		Study	
Compound	California^a^		Washington^a^	
*–*	*BE neg*	*BE pos*	*BE neg*	*BE pos*
Sugar	35.06	36.80	33.51	36.07
Added sugar	40.68	44.18	41.01	46.15
Sweetened beverages	2.10	2.26	2.81	3.13

^a^California [10] and Washington study [11] were designed and conducted, as described in the text

*BE* Barrett’s esophagus, *pos* positive for BE, *neg* negative for BE

## Discussion

The study by Li et al. [13] pooled the data of two large US studies which examined the association between sugar intake and BE. The major finding of the analysis was that the consumption of sugar containing food and beverages were positively associated with the presence of BE [13].

In line with the findings of the study by Li et al., recent investigations revealed a positive correlation between GERD and lifestyle manifestations, i. e., obesity, central obesity, intake of carbohydrates [8–12]. Conceptually sugar consumption favors obesity, which in turn stresses the geometry of the anti-reflux mechanism within the lower end of the esophagus [1, 4, 5]. As a consequence, GERD progresses. Therefore the study by Li et al. [13] extends our knowledge regarding the relevance of lifestyle to the BE. The consequences for clinical routine within our current understanding of the disease is thus still open.

GERD represents a lifestyle problem and results from the consumption of large meals, increased amount of carbohydrate-containing foods and beverages, and lack of physical activity [1–3, 8–12]. The study by Li et al. [13] clearly indicates the importance of including dietary treatment into the management of BE. Thus, in addition to adequate diagnosis, adequate control of the reflux, and elimination of BE with increased cancer risk, management should offer nutrition and lifestyle support. In addition, recent evidence confirms that an elementary diet (i. e., amino-acid-based formula, low carb diet) improves eosinophilic esophagitis, an allergic response of the esophagus [14, 15]. Therefore, diet management seems to be of importance for adequate management of GERD-related disorders of the esophagus.

Unfortunately medical therapy uses compounds (antacid drugs; proton pump inhibitors, PPI), which contain various forms of sugars (concentrated sugar, artificial sugar, sweeteners, etc.) [1, 2]. This at least may in part explain some of the side effects of PPI therapy, including gas bloat, fullness, diarrhea, and abdominal discomfort. In addition, medical treatment does not offer reflux control, it simply changes the chemical properties of the reflux, i. e., less acidic. Recent evidence indicates that less acidic reflux during PPI therapy may in fact foster the progression of BE to esophageal cancer [16]. Based on the study by Li et al. [13] and the above considerations [1, 4, 14], it seems reasonable to rethink the management of GERD and BE: the combination of an appropriate lifestyle [8–13] and effective control of reflux [1, 6, 14–16].

Lifestyle recommendations should include a significant reduction of food and beverages containing concentrated sugar, i. e., sweetener, added sugar, artificial sugars [8–13]. Following accurate diagnosis (interview, endoscopy, manometry, reflux monitoring) [1–3], the therapy should aim to eliminate the reflux by effective anti-reflux surgery (e.g., sphincter augmentation by LINX, Endostim) [1, 6]. According to the recent literature, BE with increased cancer risk should be managed by endoscopic resection (EMR) ± radiofrequency ablation (RFA) [4, 5].

Taken together, the findings of our analysis clearly show that modern management of BE has to include lifestyle measures and promote a sugar-free diet. Future studies should test how much this approach contributes to cancer prevention. May the considerations of the article foster a rethinking of current policies regarding the management of GERD and BE.
